# Editorial: Observational Methodology in Sport: Performance Key Elements

**DOI:** 10.3389/fpsyg.2020.596665

**Published:** 2020-11-12

**Authors:** Daniel Barreira, Claudio A. Casal, José L. Losada, Rubén Maneiro

**Affiliations:** ^1^Centre of Research, Education, Innovation and Intervention in Sport (CIFI2D), Faculty of Sport, University of Porto, Porto, Portugal; ^2^Physical Activity and Sports Science Faculty, Valencia Catholic University San Vicente Mártir, Valencia, Spain; ^3^Department of Social Psychology and Quantitative Psychology, University of Barcelona, Barcelona, Spain; ^4^Department of Science of Physical Activity and Sport, Pontifical University of Salamanca, Salamanca, Spain

**Keywords:** performance analysis, observational methodology, performance indicator, technical, tactical, strategic behavior

## Purpose of the Research Topic

Observational methodology is an inventive approach for performance analysis in sport that has opened up a new panorama of useful and productive research in recent years (Preciado et al.). This Research Topic (RT) responds to the need for practitioners to understand athlete and team performance in individual, dual, and team sports. This RT presents a collection of scientific articles that use observational methods, enlightening the search for performance indicators in sports, particularly how the selection and combination of Performance Key Elements (PKE) positively impact the achievement of the best performances (Brito de Souza et al.; Pérez-Turpin et al.). Additionally, the analysis of contextual variables, such as opponent level, match location or match status, provides insights into how PKE perform and consequently, impact success in sport (Valldecabres et al.).

Preciado et al. present a systematic review that provides evidence of several research lines and how they have used observational methodologies. As a rigorous and flexible scientific method, observational research allows for the analysis of spontaneous behavior in a natural context. Advances in technology and innovations in research methodology have facilitated the processes of observing, collecting, analyzing, and interpreting data, *in loco* and real-time during training sessions and competitions, as well as in *post-hoc* approaches. Strategies to measure the reliability and validity of observational instruments mean that we can perceive behaviors in different scales of performance (Lavega-Burgués et al.). We are now able to use innovative software that reduces errors and the time spent gathering data (e.g., the AMISCO system, in Fernandez-Navarro et al.), promoting novel techniques that increase possibilities of analysis (e.g., polar coordinate analysis, in Prudente et al.; or network analysis, in Praça et al.).

This RT compiles high-quality research reports of situational sports made by experts in performance analysis using observational methods to describe and interpret the PKE, preferences for tactics, and the technical and strategical structure of behavior. It is intended that these compiled papers predict the functioning of PKE in each sport, permitting practitioners to plan and design more effective play models in the future.

These papers provide readers with a greater understanding and appreciation of how PKE can be used and applied to improve athlete and team executions, with updates on current hot-topics such as performance analysis and teaching-education processes, that enable them to be better prepared to apply narrative strategies and software to analyze behaviors, improving our capacity to select and to combine different performance indicators. The compiled articles highlight opportunities to make a more specific link-to-practice regarding methodological and pedagogical innovations, permitting the creation of plans to develop the individual and collective performance in each sport.

## Overview of Contributions

A third of the 90 authors invited to contribute to this RT submitted manuscripts, evidencing the interest of the scientific community in different sports and the subject. This issue comprises 25 research articles, which include approximately 100 authors from 13 different countries with diverse research interests on observational methodology. The review processes involved over 60 experts from varied schools and specializations and four guest-editors. Studies were conducted in team sports, such as basketball, football, handball, and volleyball, in dual sports like badminton and tennis, and in individual sports like darts, combat sports, and taekwondo. These contributions examine the state-of-the-art, using theoretical and practice-driven approaches to the study of PKE and its effects in enhancing success in diverse sports.

The majority of studies discuss team sports at professional levels. In particular, football/soccer, with a systematic review by Preciado et al. about observational studies in male elite teams. Preciado et al. found that the most studied PKE were the goal, ball possession, corners, and players as individuals, mainly in national leagues and the FIFA World Cup. These data align with that on other international football leagues discussed in this RT, such as in *La Liga* (e.g., Brito de Souza et al.; González-Rodenas et al.), the *Bundesliga* (Korte et al.), and in FIFA World Cup, discussing men (e.g., Praça et al.; Clemente et al.) and women (Maneiro, Losada et al.).

Fernandez-Navarro et al. discuss *La Liga* football matches that use the AMISCO system, and found that that the winning teams in this league tended to recover more balls near their own goal, where the losing teams tended to gain possession of more balls in advanced zones of the pitch. This indicates that the better the opponent, the minor the chance of gaining the ball in advanced zones of the pitch. Awareness of the variability of these strategic spaces is also outlined by Maneiro, Blanco-Villaseñor et al. By applying the Generalizability Theory in UEFA Euro 2012 they observe that attacking the opponent allows for better exploitation of optimal parts of the playing field. The results of González-Rodenas et al. inform the attacking variables that induce greater goal-scoring opportunities, namely penetrative actions after recovering the ball and progressing by fast attacks or counterattacks. In a discussion of the Spanish Football League, Brito de Souza et al. also add that the success of teams is determined by their shooting accuracy while attacking and the number of shots conceded while defending. However, there is evidence that when playing away, the optimal PKE indicators are based on the number of shots, with defensive variables depending on the number of corners and shots conceded. Following this trend, Maneiro, Losada et al. found that in the FIFA Women World Cup 2015, successful teams had greater ball possession than unsuccessful teams, a tactical behavior that allowed them to achieve victory in most matches.

To perceive the interaction between players through passes, diverse studies have applied the Social Network Analysis. Using data from the FIFA World Cup 2018, Praça et al. and Clemente et al. searched for differences in player prominence concerning match status and positional status. On the one hand, Praça et al. found that the levels of centrality and prestige in football players in different positions indicated a more direct playing style in winning situations and a more build-up style in losing situations. On the other hand, they add that winning by an unbalanced score, significantly increased the centrality levels of the wingers and forwards in comparison to close scores. Independent of the score, defensive midfielders were the most prominent players during passing sequences. Similarly, in *Bundesliga* 1 and 2, the defensive midfielders were the main intermediary players in successful plays, but offensive midfielders were the most involved (Korte et al.).

By applying sequential analysis to the study of players' tactical numerical relations, Machado et al. found different configurations in football small-sided games (3 vs. 3+GK and 4 vs. 4+GK), where increasing rule manipulations negatively impact the exploratory behavior of youth players and teams. When discussing handball players, Prudente et al. used similar procedures of analysis and discovered that different attacking numerical relations seem to change the center back patterns of tactical behavior, more specifically in the numerical equality among the defense when there was no goalkeeper at the goal, as the center back opted for greater security and less risk of losing the ball.

Continuing the search for PKE in team sports, González-Silva et al. stated that serve criteria did not determine set efficacy in volleyball world-class top-level teams. Pérez-Turpin et al. discuss the European and World Championships 2016 and assessed PKE in women's and men's beach volleyball. They proved that there were differences in pass performance when compared among the different genders, in particular passing. In elite basketball, tactical PKE were searched by Ibáñez et al. in the Spanish Copa del Rey, and observed a greater number of attacks in the final stages of the matches, with short possessions that end in baskets or rebounds, positional attacks, and individual half-court defenses were predominant, with more shots in positional attacks and more fouls in transitions. They conclude that the style of play affects the finalization of possession. Moreover, in basketball (NBA competitions) Cui et al. studied physical PKE in relation to players' positions. They found that in addition to height and wingspan, leg power served as key determinants for being drafted as guards, as did agility and speed for power forwards and centers.

Although there are fewer publications about individual sports in this RT, a great number of sports were pointed. Bojanić et al. compared competitive combat sports with players in team sports, examining psychological PKE to conclude that self-esteem, neuroticism, and conscientiousness were the most important factors in distinguishing individual and team sports. Concerning psychological PKE, Yang et al. demonstrated that ego depletion and state anxiety have direct effects on darts sport performance curves in early-phase trials and that their interacting influence appears in late-phase trials. Two studies by Menescardi et al. about technical and tactical patterns of scoring in taekwondo, confirmed that combat sports are an important scientific Research Topic. The first study by Menescardi et al. used Markov processes and found five popular sequences: the opening and dodge, the direct attack and simultaneous counterattack, the dodge with a direct attack, the indirect attack and simultaneous counterattack, and the simultaneous counterattack with a direct attack. The second article by Menescardi et al. applied sequential analysis techniques to study two medallists in the Olympic Championships in 2012 and 2016 and concluded that athletes used the most difficult tactics to achieve the highest score.

Two studies on racket games are also included in the RT. Valldecabres et al. found significant differences in the 2015 Badminton World Championship when comparing players' on-court movements related to set, round and match status, confirming the high relevance of contextual variables in modulation of elite players' on-court movements. Gimenez-Egido et al. proposed that the Under-10 youth tennis players competition should be redesigned to build an optimal process of affordances that develop a multidimensional positive impact during this training stage. Lavega-Burgués et al. and Pic et al. also contribute to pedagogical processes in traditional sporting games, examining the 360° multimodal strategic intervention (decisional, relational, and organic) in a Mario game using t-pattern analysis, and concluding that it is of high importance for the development of a child. With the same procedures, Pic et al. confirmed the differences between genders also found by Pérez-Turpin et al. in their paper on elite beach volleyball. Pic et al. studied the differences between boys and girls while playing Triadic Motor Games, observing more diverse motor solutions in girls, while the boys' behavior showed a greater specialization in roles and sub-roles and the association of these solutions with a favorable modification of the marker. Lavega-Burgués et al. and Pic et al. present key findings in improving physical education teaching-learning processes, contributions to the diversity of knowledge, values, and attitudes necessary for society today.

## Recommendations for Future Research

### The Importance of Analyzing the Player

Recent systematic reviews (e.g., Sarmento et al., 2014) have revealed that there is limited research on the individual parameters of performance in players, confirmed other studies in this RT. Nevertheless, recent research provides significant insights on the individual indicators in players, for example, Yi et al. (2020) studied associations among the variations of technical indicators, playing positions, and situational variables. Castañer et al. (2016) analyze individual patterns of play in top-level players. Finally, Castañer et al. (2017) compared elite players' motor and technical skills. Despite these contributions, there few studies have related these findings to all the dimensions of performance, namely the physical, tactical, and psychological characteristics of players.

It is vital to improve observational instruments and software to uncover how the players: (i) perform visual exploratory activity in game contexts (McGuckian et al., 2018); (ii) develop technical and tactical skills over time, using longitudinal studies (Yi et al., 2020); and (iii) manage functional asymmetry as a control of performance in relation to global performance dimensions (Guilherme et al., 2015). Technical skills assessment also needs to be further developed in the natural contexts of sport, for example, small-sided games in football, by validating observational instruments that permit us to extract data during matches or in other contextualized backgrounds. As the majority of studies emphasize the attacking phase of the game, we also advise authors to, in the future, provide more detail about defending behaviors (Fernandes et al., 2020).

### To Improve Teams' Skills

Teams that work on professional levels have been studied in detail over the years, however taking into consideration isolated collective PKE, we encourage researchers to investigate time, space, and task macro-structures and the numerical relations of both teams (Perl and Memmert, 2017). In other words, it is important to comprehend how teams manage the time and the quality of skills at their disposal simultaneously, and how they gain advantages in the playing space through interrelating positions along with their orientation to the ball (Yi et al., 2020). Defending plays (Fernandes et al., 2020) and set-pieces need to be studied more in the future to better inform practitioners about effective behaviors in phases of the game. The performances of youth teams may also be further explored in future research perspectives.

### Insights Into Training and Teaching-Learning Processes

We suggest that future researchers advance the development of educational specific processes (e.g., Machado et al.) by answering the following question: “*how and when do the behaviors can be better trained and learned?*”. We advise them to develop novel and concrete pedagogical principles and methodologies, testing them in longitudinal studies and exploring: how non-linear pedagogy can improve individual and collective PKE; when and how structured training starts in youth athletes; which team disposition of play better improves different players' individual characteristics (e.g., Memmert et al., 2019); how the opponent level in individual and team sports can create challenges that improve the development of players.

## Conclusion

The scientific articles included in this RT focus on research questions that progress the field of performance analysis and guide future research on which (combination of) factors may be studied to enhance insight in sports. The stimulating research designs show novel possibilities of analysis, offering understandings of new research questions, and how they may be studied, developing new ideas about how to apply scientific knowledge in practice of diverse sports.

## Author Contributions

DB and CC contributed to the manuscript review and wrote the first draft. JL and RM reviewed the manuscript. All authors contributed to the article and approved the submitted version.

## Conflict of Interest

The authors declare that the research was conducted in the absence of any commercial or financial relationships that could be construed as a potential conflict of interest.
